# Development of a Methodology for Analyzing Microplastics in Sewage Treatment Plants

**DOI:** 10.1007/s00244-026-01208-2

**Published:** 2026-07-21

**Authors:** Ivanilson da Silva de Aquino, Otilie Eichler Vercillo, Wellington Marcos da Silva, Alisson Mendes Rodrigues, Ariuska Karla Barbosa Amorim

**Affiliations:** 1 Department of Civil and Environmental Engineering, University of Brasilia (UnB), Asa Norte, Brasilia, DF 70910-900 Brazil; 2 UnB Planaltina Faculty, University of Brasilia (UnB), Brasilia, DF 70904-910 Brazil; 3 Innovative Materials Solutions Group (SIM), Institute of Engineering, Science and Technology (IECT), Federal University of Jequitinhonha and Mucuri Valleys (UFVJM), Av. Um, 4050, Cidade Universitária, Janaúba, MG 39447-790 Brazil

## Abstract

Global pollution by MPs has become an emerging concern, and recent studies have focused on analyzing their presence in wastewater treatment systems. One of the main challenges in this field is the absence of standardized methods, reference materials, and comparative data. This study aimed to develop, optimize, and validate a methodological framework for the extraction and identification of MPs in wastewater matrices. Reference MPs were produced from the polymers such as polyethylene terephthalate (PET), high-density polyethylene (HDPE), low-density polyethylene (LDPE), polyvinyl chloride (PVC), polypropylene (PP), and polystyrene (PS). To evaluate the integrity of these MPs after digestion, five Fenton reagent protocols were tested, and the carbonyl index was applied. For density-based separation, sodium chloride (NaCl) and zinc chloride (ZnCl_2_) solutions at different concentrations were used in a density separation device. The MPs obtained presented irregular fragments ranging from 0.1 to 3.0 mm. FTIR analysis performed before and after Fenton digestion showed no significant spectral deviations. The optimal Fenton conditions involved temperatures between 40 and 60 °C and 2 h of reaction time. ZnCl_2_ provided the best performance for particle recovery, ensuring high separation efficiency. The optimized methodology was subsequently validated using raw wastewater and dewatered sewage sludge collected from a full-scale WWTP. The validation demonstrated effective organic matter removal, successful recovery of spiked MPs, and reliable identification of native MPs present in the environmental samples. Overall, the proposed methodology proved to be a robust and reliable approach for the extraction and characterization of MPs in complex wastewater matrices, providing methodological support for future monitoring programs and contributing to the development of standardized protocols for MPs analysis.

## Introduction

The continuous growth in global plastic manufacturing and consumption has resulted in the widespread accumulation of plastic residues across natural ecosystems. Through continuous exposure to physical, chemical, and biological weathering, larger plastic debris progressively breaks down into smaller particles known as microplastics (MPs), which are now widely recognized as persistent environmental contaminants with potential ecological implications (Corcoran 2022). MPs are solid polymeric particles generally smaller than 5 mm that originate either from the intentional production of microscopic plastic materials, known as primary MPs, or from the progressive fragmentation of larger plastic objects through physical, chemical, and biological weathering processes, referred to as secondary MPs (Ratnayake et al. 2024; Mishra et al. 2025). Due to their small size (< 5 mm), they present high dispersion capacity and resistance to natural manipulation and are persistent and bioaccumulative across a wide range of ecosystems (de Sá et al. 2018; Campanale et al. 2020). Although research on the occurrence of MPs in environmental matrices has expanded considerably in recent decades, the development of standardized protocols for their sampling, extraction, and identification remains limited and technically challenging, particularly in wastewater matrices. These challenges are mainly associated with the high complexity of sewage samples, which contain elevated concentrations of organic matter, inorganic particles, biological residues, suspended solids, and potential contaminants that can interfere with MP recovery, separation efficiency, and polymer identification.

MPs enter the environment through multiple pathways, including surface runoff, effluent discharge from wastewater treatment plants (WWTPs) and industrial facilities, leaching from sewage sludge applied to agricultural soils or disposed of in landfills, and atmospheric deposition (Al-Amri et al. 2024). Among these pathways, WWTPs are considered major conduits for the transfer of MPs to aquatic environments, as they receive large volumes of domestic and industrial wastewater containing plastic debris. These units have been identified as primary sources of MPs in aquatic environments, releasing these particles both through treated effluents and through sludge-derived products (Murphy et al. 2016; Sun et al. 2019; Long et al. 2019). Furthermore, MPs extracted from wastewater are incorporated into sewage sludge, which constitutes another problem due to their application in agricultural areas (Zubris and Richards 2005; Cydzik-Kwiatkowska et al. 2022).

The investigation of MPs in WWTPs has therefore become an important area of research, requiring reliable analytical approaches for their detection, extraction, and identification. One of the main challenges in this field is the complexity of environmental matrices, in which MPs are often associated with organic matter, inorganic particles, and biological residues. The removal of these interfering materials prior to analysis is a critical step to ensure accurate quantification and reliable identification of MPs (Badzoka et al. 2025). To isolate microplastics from complex environmental matrices, several pretreatment strategies have been proposed, including acid, base, and oxidative digestion, as well as enzymatic treatments to remove organic matter. Each of these approaches presents advantages and limitations regarding digestion efficiency, operational costs, and preservation of polymer integrity. Among these methods, Fenton's reagent has received considerable attention for its strong oxidative capacity and ability to degrade organic matter while potentially maintaining the structural integrity of plastic (Li et al. 2020; Kang et al. 2020; Lavoy and Crossman 2021). Furthermore, density separation of MPs using saline solutions is widely used for particle recovery and preconcentration, and salts such as NaCl, ZnCl2, and ZnBr2 have been investigated to optimize separation based on density and polymer type (Quinn et al. 2017). Advanced tools such as Fourier transform infrared spectroscopy (FTIR), Raman spectroscopy, pyrolysis coupled with gas chromatography–mass spectrometry (Pyr-GC–MS), and scanning electron microscopy (SEM) are usually required for accurate characterization of MPs (Hidalgo-Ruz et al. 2012).

The present study proposes and evaluates a methodological framework for the extraction and identification of MPs in WWTPs. The approach includes the production reference MPs from common commercial polymers, evaluation of polymer resistance to chemical digestion using Fenton's reagent through FTIR analysis and Carbonyl Index assessment, and the investigation of density-based separation efficiency using different saline solutions. In addition, the optimized protocol was validated using real wastewater matrices, including raw wastewater and dewatered sewage sludge collected from a full-scale WWTP, to assess its applicability under realistic environmental conditions. The validation involved evaluating organic matter removal efficiency, MPs recovery, and polymer identification after sample processing. By combining laboratory optimization with validation in complex environmental matrices, the proposed methodology provides a reliable protocol for the extraction, recovery, and identification of MPs while preserving polymer integrity, thereby contributing to a better understanding of the occurrence, transport, and fate of MPs in wastewater treatment systems.

## Materials

All chemicals employed in this study were of analytical grade. Ferrous sulfate heptahydrate (FeSO_4_·7H_2_O, Sigma-Aldrich, 99%), hydrogen peroxide (H_2_O_2_, Merck, 30%) and sulfuric acid (H_2_SO_4_, Sigma-Aldrich, 98%), sodium chloride (NaCl, Vetec, 99%), and zinc chloride (ZnCl_2_, Dinamica, 98%) were used without additional purification.

### Production of Reference MPs

Reference MPs were generated from six commercial plastic products purchased from a local market. The selected polymers included PET (beverage bottles), HDPE (detergent containers), LDPE (plastic bags), PVC (water pipes), PP (food containers), and PS (disposable cups). To simulate environmentally relevant fragments, the materials were initially cut into pieces measuring approximately 3–5 cm using stainless steel scissors and subsequently ground at 12,000 rpm for 5 min. The resulting particles were sieved to obtain sizes of ≤ 3 mm. Due to mechanical fragmentation, the particles exhibited irregular shapes, as is common in environmental samples. Each polymer was processed individually to avoid cross-contamination.

### Characterization Techniques

The chemical composition of the microplastics was characterized by Fourier Transform Infrared (FTIR) spectroscopy analyses were performed using a PerkinElmer Frontier MIR + SP10 STD spectrometer (PerkinElmer, Waltham, MA, USA), equipped with an Attenuated Total Reflectance (ATR) accessory featuring a Zinc Selenide (ZnSe) crystal. Spectra were collected over the range 4000 to 650 cm^−1^, with a resolution of 4 cm^−1^ and 64 scans, using powdered MP samples. FTIR spectra were obtained both before and after the digestion protocols. The morphology of the MPs was examined using a digital stereomicroscope (Leica EZ4 D, Leica Microsystems, Wetzlar, Germany).

### Evaluation of Polymer Chemical Resistance to Fenton’s Oxidation

Five different digestion protocols were applied to each MP separately to assess its chemical resistance to Fenton's reagent. Each protocol was characterized by a specific temperature and reaction time, as shown in Table 1. The selected conditions were based on oxidation parameters commonly reported in the literature and were designed to represent mild, intermediate, and severe digestion conditions, allowing the evaluation of both the efficiency of organic matter degradation and the preservation of polymer integrity after treatment. Initially, a FeSO_4_·7H_2_O solution (278 gmol^−1^) was added to 500 mg of MPs, followed by the addition of 3 mL of concentrated H_2_SO_4_. Subsequently, an additional 20 mL aliquot of the FeSO_4_·7H_2_O solution and 20 mL of H_2_O_2_ (30% w/w) were introduced into the system. The reaction mixture was then maintained at the predetermined temperature and for the reaction time specified for each digestion protocol.

**Table 1:** Experimental conditions (temperature and time) for the Fenton’s reagent digestion protocols

Fenton digestion protocol	Temperature (°C)	Time (h)
1	RT^a^	24
2	40	2
3	60	2
4	80	2
5	100	2

^a^room temperature

The carbonyl index (CI) was calculated from the FTIR spectra using Eq. 1. It corresponds to the ratio between the absorbance of the carbonyl region (1715–1735 cm^−1^) and a characteristic reference band for each polymer: 1471 cm^−1^ for PE (HDPE and LDPE), 1458 cm^−1^ for PP, 1452 cm^−1^ for PS, 1508 cm^−1^ for PET, and 1328 cm^−1^ for PVC (Balakit et al. 2015; Rodrigues et al. 2018).

1 $$\mathrm{C}\mathrm{I}=\frac{\mathrm{B}\mathrm{a}\mathrm{n}\mathrm{d} 1715-1735 {\mathrm{c}\mathrm{m}}^{-1}}{{\mathrm{R}\mathrm{e}\mathrm{f}\mathrm{e}\mathrm{r}\mathrm{e}\mathrm{n}\mathrm{c}\mathrm{e} \;\mathrm{b}\mathrm{a}\mathrm{n}\mathrm{d}\; \mathrm{c}\mathrm{m}}^{-1}}$$

### Design and Operation of the Custom Density Separation Unit

**Fig. 1:**
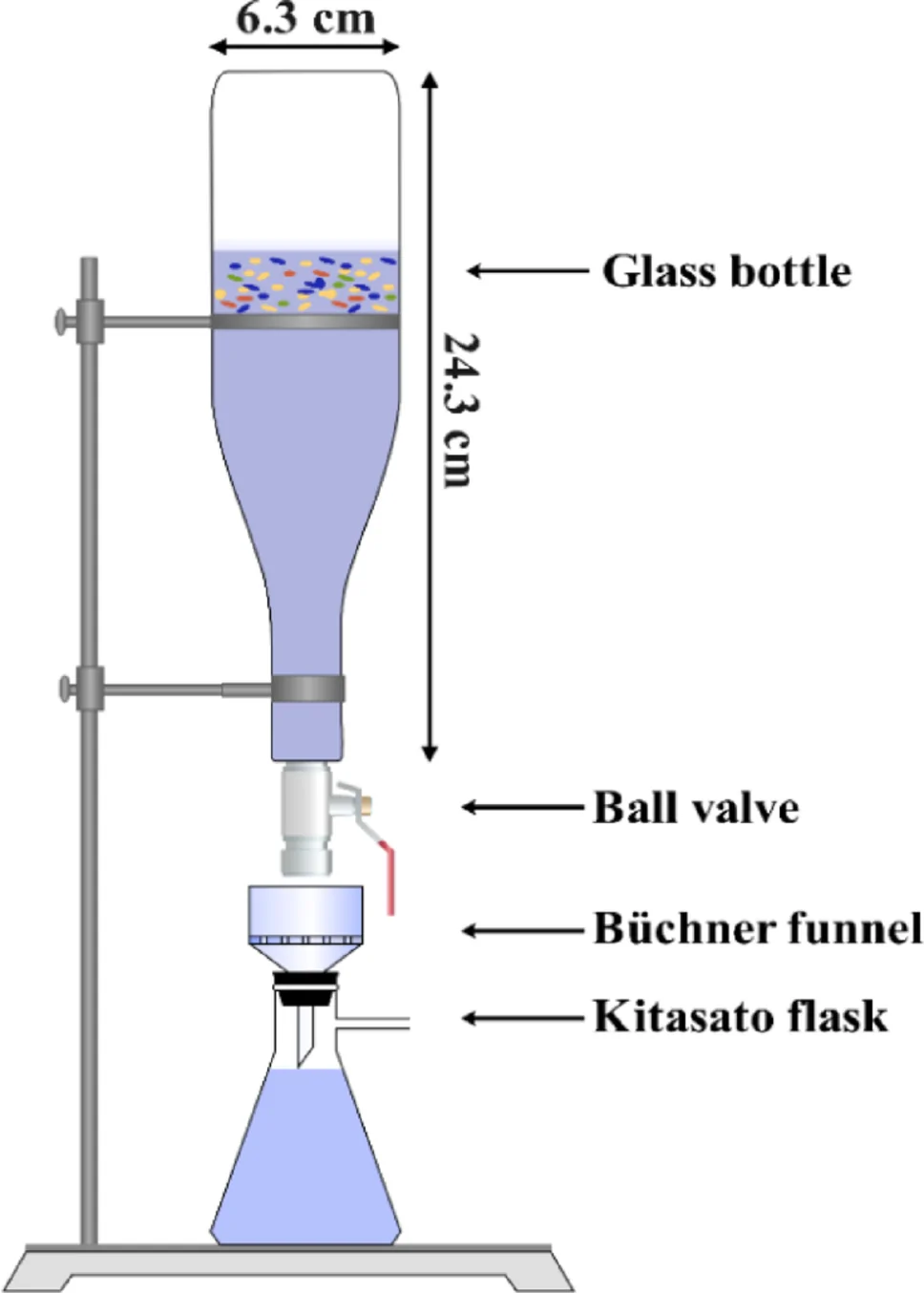
Schematic representation of the customized glass separation unit designed for density-based extraction of MPs

To determine the efficiency of the isolation procedure, recovery tests of MPs were performed using a density-based separation approach. This approach relies on the density contrast between polymer particles and the surrounding medium, allowing microplastics to be separated from complex environmental matrices (Han et al. 2019). Based on a review of existing protocols, a compact extraction unit was designed to prioritize simplicity, speed, and efficiency while minimizing cross-contamination risks. The device consisted of a glass vessel equipped with a stainless-steel barbed fitting and a ball valve, enabling the direct decantation of the denser sedimented fraction, while the MP-containing supernatant remains in the chamber for subsequent collection (Fig. 1). Glass was selected for the unit’s construction because its smooth, non-porous surface facilitates particle flow and prevents MP retention. In addition, the use of glassware is commonly recommended in MPs analysis to minimize contamination from synthetic fibers and polymer fragments that may originate from plastic laboratory equipment (Aminah and Ikejima 2023).

A series of brine solutions was obtained by dissolving sodium chloride or zinc chloride in 1000 mL of distilled water in order to obtain density values ranging from 1.2 to 1.8 g cm⁻^3^. Each solution was prepared in a 2 L glass beaker and mixed with a magnetic stirrer for approximately 30 min to promote complete salt dissolution and ensure homogeneity of the density medium. Details regarding the salts employed in the preparation of the density solutions are summarized in Table 2. An aliquot of 250 mL of the prepared saline solution was transferred to the custom separation unit, and 60 polymer discs (10 units per polymer type) were added. The mixture was vigorously stirred with a glass rod to ensure thorough wetting and contact between the density medium and the particles, then left undisturbed for 1 h to allow gravitational separation. The sedimented fraction was recovered by rapidly opening the ball valve and discharging it into a Büchner funnel equipped with a glass-fiber membrane filter (Merck Millipore, 47 mm diameter, 0.7 µm pore size, 475 µm thickness) for filtration and subsequent particle counting. The remaining floating fraction was similarly recovered to calculate the total mass balance. This procedure was performed in triplicate for each solution tested.

**Table 2:** Formulation of NaCl and ZnCl_2_ brine solutions used in the MPs recovery experiments

Salt	Density (g cm^−3^)	Amount added to 1 L H_2_O (g)
NaCl	1.2	337
ZnCl_2_	1.3	500
ZnCl_2_	1.6	972
ZnCl_2_	1.8	1800

### Validation of the Proposed Methodology Using Real Wastewater Matrices

To evaluate the applicability of the proposed methodology under realistic conditions, validation experiments were conducted using raw wastewater and dewatered sewage sludge collected from the Brasília North Wastewater Treatment Plant (Brasília North WWTP), operated by the Federal District Environmental Sanitation Company (CAESB), located in Brasília, Brazil's capital. It treats an average flow of 38,880 m^3^/day and serves a population of approximately 260,000 inhabitants. The sewage treatment system adopted for Brasília North WWTP consists of preliminary treatment (coarse screening with mechanical cleaning and fine screening with mechanical cleaning to remove coarse solids), desanders, primary decanters, activated sludge with removal of carbon, nitrogen and phosphorus, and final polishing (coagulation/flocculation process and dissolved air flotation). Raw wastewater samples were obtained from the influent channel prior to any treatment process, whereas the sludge samples consisted of dewatered sludge collected after the sludge treatment and dewatering stages, representing the final sludge produced by the WWTP. Samples were collected in pre-cleaned containers (600 mL), transported to the laboratory under refrigerated conditions (4 °C), and processed within 24 h of collection. Prior to the validation experiments, the samples were thoroughly homogenized to ensure representative subsampling (magnetic stirring, 300 rpm, 10 min).

The use of both matrices allowed the evaluation of the proposed methodology under different operational conditions commonly encountered in WWTPs. Raw wastewater was selected to assess the performance of the method in the liquid phase of the treatment process, where MPs interact with suspended solids, organic matter, and microbial communities. Dewatered sewage sludge was included because it represents one of the most complex matrices generated during wastewater treatment, characterized by elevated concentrations of organic matter, microbial biomass, and inorganic solids. Therefore, the successful recovery of MPs from both matrices provides evidence of the applicability of the proposed methodology across different stages of wastewater treatment systems.

Prior to the validation experiments, aliquots of raw wastewater and dewatered sewage sludge were processed separately to assess the presence of native MPs and to minimize potential interference during the interpretation of recovery results. A known quantity of reference MPs, consisting of PET, HDPE, PVC, LDPE, PP, and PS particles previously characterized by stereomicroscopy and FTIR, was then added to aliquots of raw wastewater and dewatered sewage sludge.

To evaluate the recovery efficiency of the proposed methodology, validation experiments were performed using wastewater-derived matrices enriched with reference MPs. Raw wastewater samples were initially processed through a set of stainless-steel sieves (5 mm to 45 μm, ASTM standard), and the retained material was used as the representative wastewater matrix. For the sludge matrix, a representative aliquot of 10 g (wet weight) of dewatered sewage sludge was used. A total of 60 reference MPs were added to each matrix, consisting of 10 particles of each polymer type (PET, HDPE, PVC, LDPE, PP, and PS). The particles had been previously characterized by stereomicroscopy and ATR-FTIR. After enrichment, the samples were homogenized to ensure adequate contact between the MPs and the surrounding organic matrix and maintained under static conditions for 24 h at room temperature. This procedure was adopted to promote the adhesion of suspended solids, organic matter, and microorganisms onto the MPs surface, simulating conditions commonly encountered in wastewater treatment systems. Subsequently, the samples were subjected to the optimized Fenton digestion protocol established in this study at the selected temperature and reaction time (see Table 2). The objective of this step was to reduce the organic matter content while preserving the physicochemical integrity of the MPs and maintaining their suitability for subsequent density separation and FTIR identification.

### Evaluation of Organic Matter Removal Efficiency

The efficiency of organic matter removal (OMR%) during Fenton digestion was quantitatively evaluated using chemical oxygen demand (COD) analyses. COD measurements were performed on raw wastewater and sewage sludge samples, before and after digestion, according to the Standard Methods for the Examination of Water and Wastewater (APHA; AWWA; WEF 2017). For raw wastewater, aliquots of 2.0 mL were directly transferred to COD digestion vials. For dewatered sewage sludge, samples were initially homogenized with ultrapure water at a ratio of 1:10 (w/v) and subsequently diluted when necessary to ensure that COD values remained within the analytical range of the method. Aliquots were collected immediately prior to the addition of Fenton's reagent and after completion of the digestion process. COD analyses were performed using Hach COD digestion vials and a DR2800 spectrophotometer (Hach Company, Loveland, CO, USA), following the manufacturer's instructions. The percentage removal of organic matter was calculated according toto Eq. 2:

2 $$OMR \left(\%\right)=\left[\frac{\left(CO{D}_{0}-CO{D}_{f}\right)}{CO{D}_{0}}\right].100$$

where COD₀ is the initial COD concentration and COD_f_ is the COD concentration after Fenton digestion. The reduction in COD was used as an indicator of the effectiveness of the digestion procedure in removing organic matter from complex wastewater matrices.

### Recovery of MPs Following Digestion

Following digestion, the resulting suspensions were subjected to density separation using ZnCl₂ solution (1.8 g cm^−3^). This density medium was selected because it enables the flotation of both low-density and high-density polymers, including PET and PVC. The samples were homogenized (magnetic stirring, 500 rpm, 10 min) and allowed to settle for 24 h to promote gravitational separation between floating MPs and the denser sediment fraction. The floating fraction was subsequently recovered and filtered through a glass fiber membrane filter (Merck Millipore, 47 mm diameter, 0.7 µm pore size, 475 µm thickness).

The retained particles were examined using a stereomicroscope and subsequently identified by FTIR-ATR to verify their recovery and evaluate potential alterations resulting from the pretreatment procedures. The performance of the proposed methodology was assessed based on MP recovery, reduction of organic matter content, and preservation of the characteristic FTIR spectral features required for polymer identification.

### Application of the Validated Methodology to Native MPs

After validation using wastewater and sludge samples enriched with reference MPs, the optimized methodology was applied to environmental samples without the addition of MPs. Raw wastewater and dewatered sewage sludge collected from the Brasília North WWTP were subjected to the same sequence of digestion, density separation, filtration, and identification procedures described above. For raw wastewater samples, an initial volume of sample was passed through a stainless-steel sieve cascade composed of circular ASTM sieves with mesh sizes ranging from 5 mm to 45 μm. This step allowed the retention and concentration of particulate material potentially containing MPs while removing excess water. The material retained on the sieves was subsequently transferred to glass containers for further processing. For dewatered sewage sludge, a representative aliquot of 10 g (wet weight) was collected and homogenized prior to analysis. The sludge samples were then subjected directly to the digestion procedure. The recovered particles were examined under a stereomicroscope, and representative particles exhibiting morphologies typically associated with environmental MPs, including fragments, films, and fibers, were selected for FTIR-ATR analysis.

### Quality Control

During the laboratory procedure, several measures were adopted to minimize possible cross-contamination by other sources of MPs. Glassware was preferably used, avoiding the use of plastic materials. In addition, a cotton lab coat and nitrile gloves were used, and a filter membrane sheet was also used on the table to assess MPs pollution in the air. The laboratory containers were rinsed three times with distilled water and always covered with aluminum foil during the sample processing steps. Two blank experiments were performed throughout the MP analysis process, and the results revealed no external MPs pollution, indicating an analysis environment free of MPs particles under the conditions evaluated.

## Results and Discussions

### Morphology and FTIR Characterization Before Digesting Protocols

**Fig. 2:**
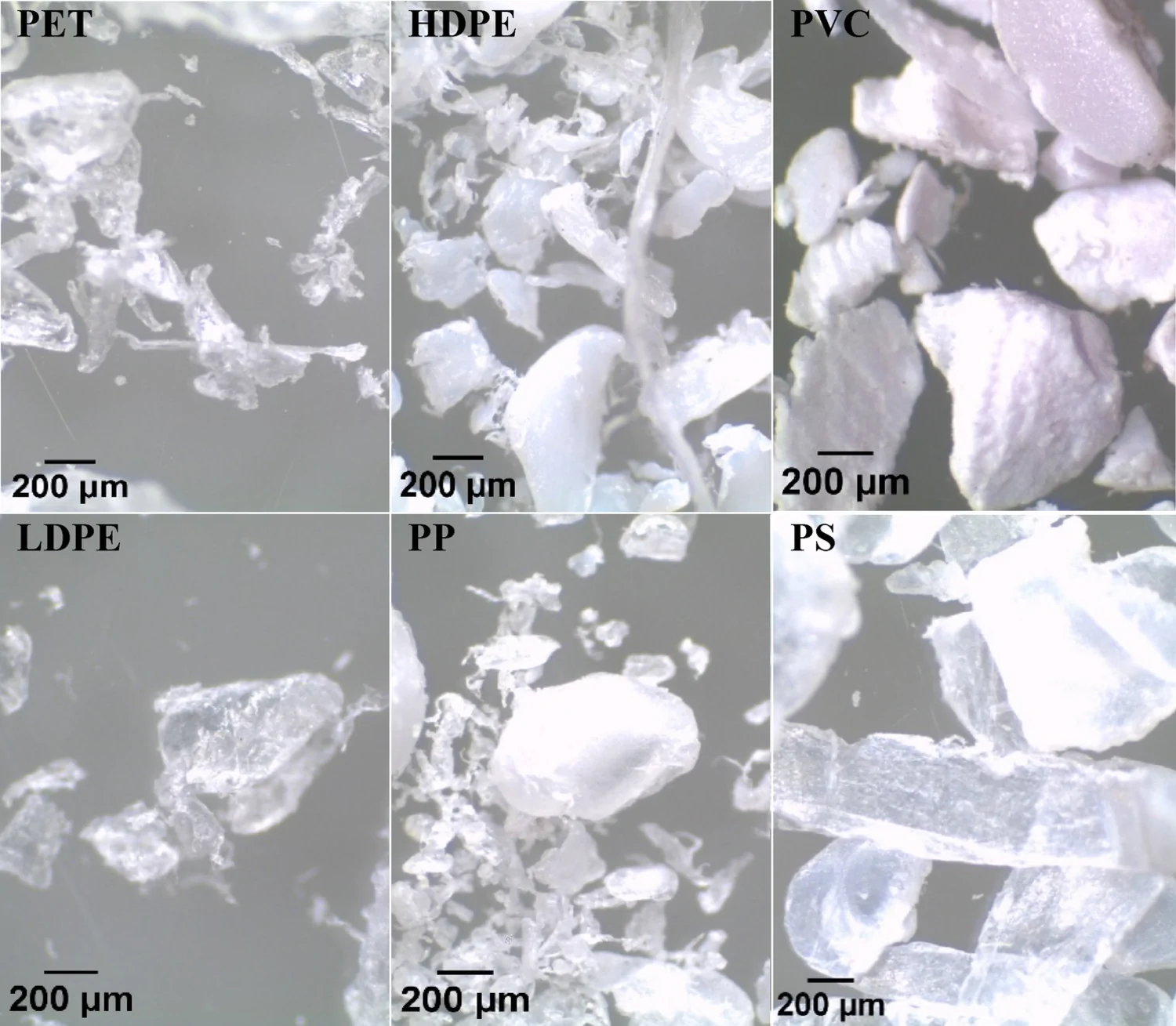
Stereomicroscope images of the six target MPs (PET, HDPE, PVC, LDPE, PP, and PS), showing varied sizes and irregular surface morphologies

**Fig. 3:**
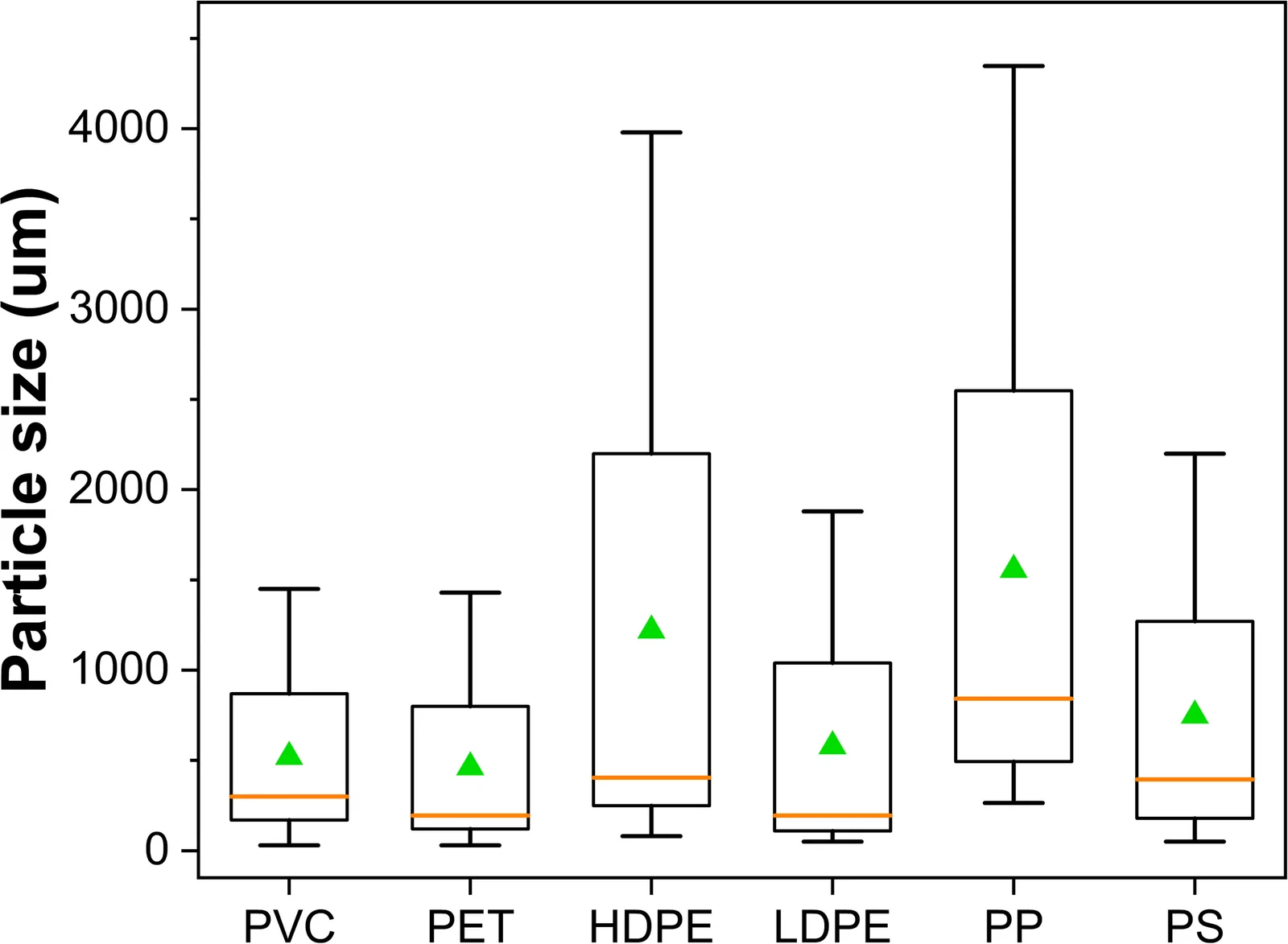
Particle size distribution of MPs obtained from commercial polymers (PVC, PET, HDPE, LDPE, PP, and PS). Box plots represent the interquartile range (Q1–Q3), with the orange horizontal line indicating the median particle size. Whiskers represent the minimum and maximum observed values, and green triangles denote the mean particle size

The MPs obtained from the commercial polymers (PET, HDPE, PVC, LDPE, PP, and PS) are shown in Fig. 2. All samples presented heterogeneous shapes, predominantly with rough surfaces resulting from grinding. Particle size distributions, determined via image analysis, are presented in Fig. 3. PVC and PET showed relatively narrow distributions, with median sizes below 500 µm and maximum values near 1500 µm. In contrast, LDPE and PS showed broader distributions; while their medians were around 300 µm, their upper extremes reached nearly 2000 µm and 2200 µm, respectively. HDPE and PP presented the widest ranges, with medians between 400 and 700 µm. Although samples were sieved at 3 mm, some particles with elongated geometries presented lengths up to ~ 4000 µm (HDPE) and 4500 µm (PP) along their major axis. These results highlight the morphological heterogeneity of the produced MPs, consistent with simulated environmental degradation and significantly influencing surface area, reactivity, and particle buoyancy during density separation.

**Fig. 4:**
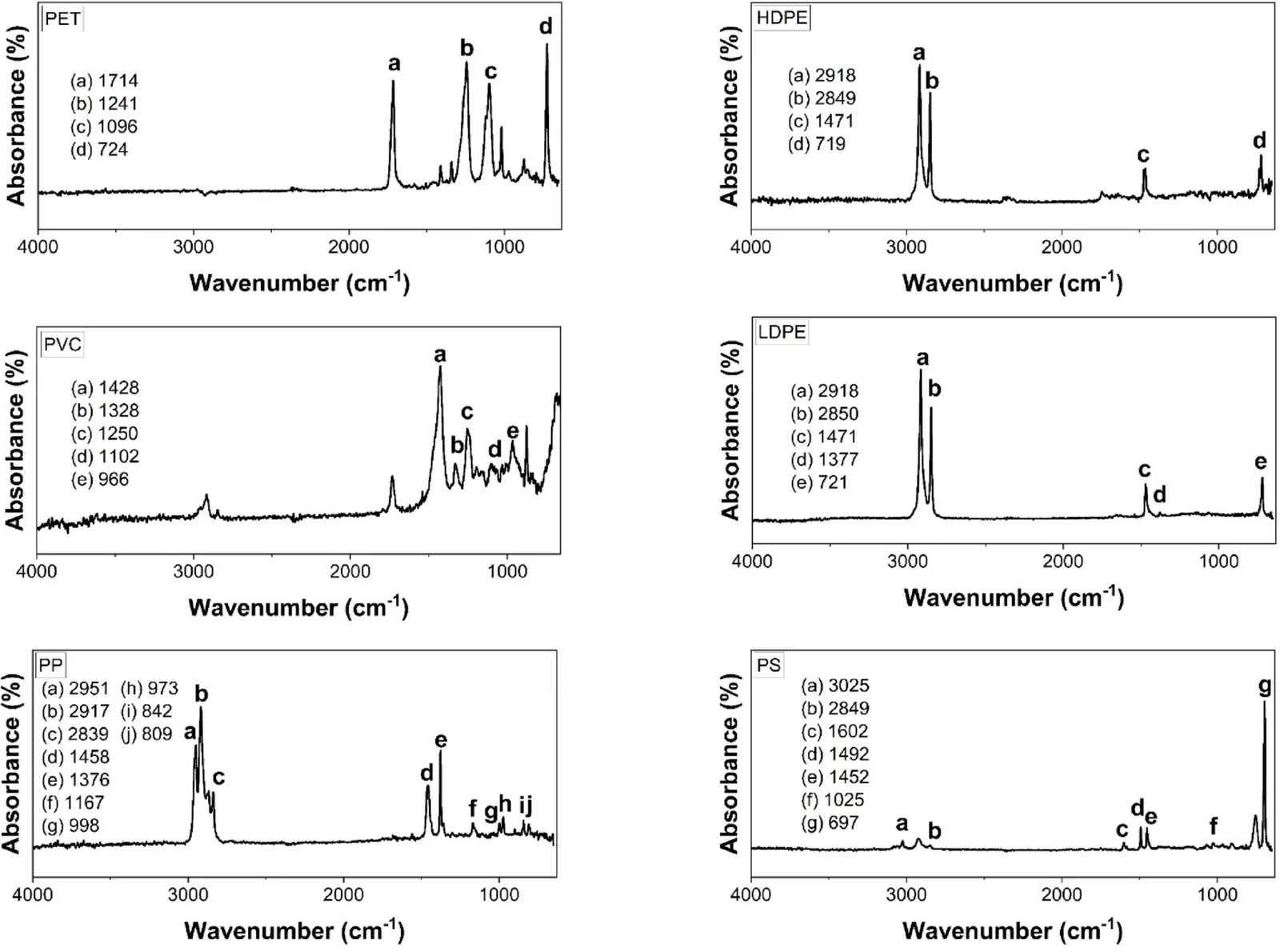
FTIR-ATR spectra of the MPs obtained from the PET, HDPE, PVC, LDPE, PP and PS

The infrared spectra of PET, HDPE, PVC, LDPE, PP, and PS are shown in Fig. 4 displayed characteristic absorption features consistent with their respective polymer structures. A comparison with reference spectra reported in the literature (Table 3) confirmed the identity of the materials analyzed. The positions of the main absorption bands were in close agreement with previously reported values (Chércoles Asensio et al. 2009; Jung et al. 2018a), with minor shifts not exceeding four wavenumbers, which can be attributed to differences in experimental conditions and sample morphology.

**Table 3:** Comparison of absorption bands obtained via ATR-FTIR from MPs with literature studies

Polymer	This work	Characteristic peaks (cm^−1^)	Assignment	References
HDPE	(a) ~ 2918 (b) ~ 2849 (c) ~ 1471 (d) ~ 719	(a) ~ 2915 (b) ~ 2845 (c) ~ 1472 (d) ~ 717	(a) Stretching C–H (b) Stretching C–H (c) Flexion CH_2_ (d) Balance sheet CH_2_	Chércoles Asensio et al. (2009), Jung et al. (2018a)
LDPE	(a) ~ 2918 (b) ~ 2850 (c) ~ 1471 (d) ~ 1377 (e) ~ 721	(a) ~ 2915 (b) ~ 2845 (c) ~ 1467 (d) ~ 1377 (e) ~ 717	(a) Stretching C–H (b) Stretching C–H (c) Flexion CH_2_ (d) Flexion CH_3_ (e) Balance sheet CH_2_	Chércoles Asensio et al. (2009), Jung et al. (2018a)
PET	(a) ~ 1714 (b) ~ 1241 (c) ~ 1096 (d) ~ 724	(a) ~ 1713 (b) ~ 1241 (c) ~ 1094 (d) ~ 720	(a) Stretching C=O (b) Stretching C–O (c) Stretching C–O (d) Out-of-plane bending Aromatic CH	Chércoles Asensio et al. (2009), Jung et al. (2018a)
PP	(a) ~ 2951 (b) ~ 2917 (c) ~ 2839 (d) ~ 1458 (e) ~ 1376 (f) ~ 1167 (g) ~ 998 (h) ~ 972 (i) ~ 842 (j) ~ 809	(a) ~ 2950 (b) ~ 2915 (c) ~ 2838 (d) ~ 1455 (e) ~ 1377 (f) ~ 1166 (g) ~ 997 (h) ~ 972 (i) ~ 840 (j) ~ 808	(a) Stretching C–H (b) Stretching C–H (c) Stretching C–H (d) Flexion CH_2_ (e) Flexion CH_3_ (f) Flexion CH, Balance sheet CH_3_ (g) Stretching C–C (h) Balance sheet CH_3_, Flexion CH_3_, Flexion CH (i) Balance sheet CH_3_, Stretching C–C (j) Balance sheet CH_2_, Stretching C–CH_3_	Chércoles Asensio et al. (2009), Jung et al. (2018a)
PS	(a) ~ 3025 (b) ~ 2849 (c) ~ 1602 (d) ~ 1492 (e) ~ 1452 (f) ~ 1025 (g) ~ 697	(a) ~ 3024 (b) ~ 2847 (c) ~ 1601 (d) ~ 1492 (e) ~ 1451 (f) ~ 1027 (g) ~ 694	(a) Stretching C–H aromatic (b) Stretching C–H (c) Stretching of the aromatic ring (d) Stretching of the aromatic ring (e) Flexion CH_2_ (f) Aromatic CH folding (g) Aromatic CH out-of-plane curve	Chércoles Asensio et al. (2009), Jung et al. (2018a)
PVC	(a) ~ 1428 (b) ~ 1328 (c) ~ 1250 (d) ~ 1102 (e) ~ 966	(a) ~ 1427 (b) ~ 1331 (c) ~ 1255 (d) ~ 1099 (e) ~ 966	(a) Flexion CH_2_ (b) Flexion CH (c) Flexion CH (d) Stretching C–C (e) Balance sheet CH_2_	Chércoles Asensio et al. (2009), Jung et al. (2018a)

### Integrity of MPs After the Chemical Digestion Process

**Fig. 5:**
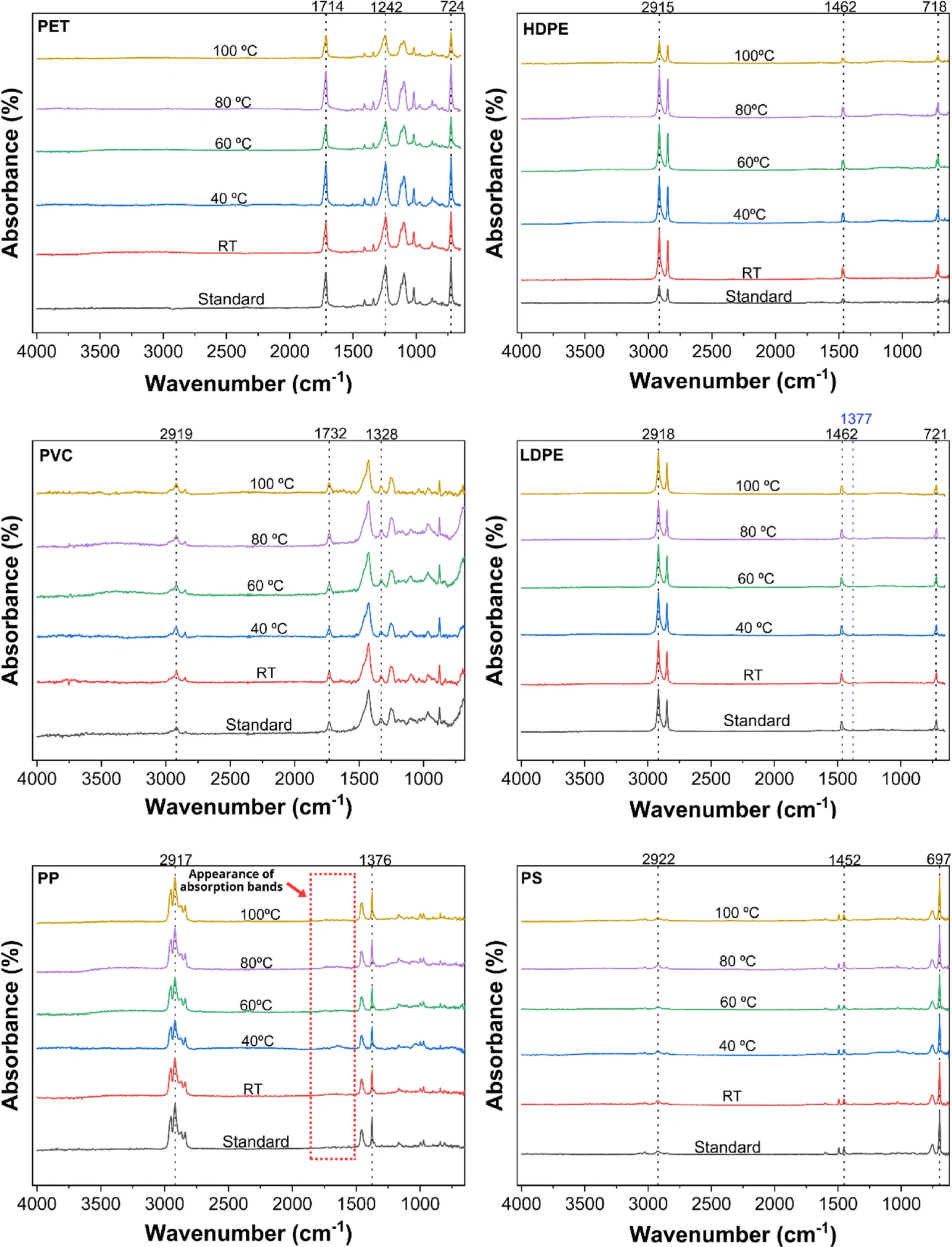
FTIR spectra of MPs for the different digestion protocols employed

FTIR-ATR spectra of the MPs obtained before (Standard) and after (RT, 40–100 °C) Fenton-reaction digestion protocols are shown in Fig. 5. The influence of the digestion procedures was assessed based on changes in the characteristic absorption bands used for polymer identification. The main peaks shown in Fig. 5 were 1714, 1242, and 724 cm^−1^ for PET; 718, 1462, and 2915 cm^−1^ for HDPE; 1328, 1732, and 2919 cm^−1^ for PVC; 721, 1377, 1462, and 2918 cm^−1^ for LDPE; 1376 and 2917 cm^−1^ for PP; and 697, 1452, and 2922 cm^−1^ for PS. Changes in the intensity or appearance of these bands were used to evaluate possible structural alterations induced by the digestion process.

After the Fenton reaction digestion protocols, the FTIR spectra did not show significant deviations from the initial spectra (without digestion), allowing the identification of the polymers. Hurley et al., (2018) evaluated four chemical digestion protocols and their influence on the identification of MPs by FTIR (Hurley et al. 2018). The application of the Fenton reaction did not result in significant alterations in the FTIR spectral features of the analyzed polymers, including PP, LDPE, HDPE, PS, PET, polyamide 6.6 (PA-6.6), polycarbonate (PC), and polymethyl methacrylate (PMMA). These findings indicate that Fenton digestion does not compromise the chemical characterization of MPs by FTIR-ATR, as the main absorption bands required for polymer fingerprint identification remain preserved. However, although FTIR is widely used to evaluate the effects of pretreatment methods on MPs, relying solely on qualitative spectral analysis may not provide a comprehensive understanding of structural changes induced by digestion processes. Following Fenton digestion, the FTIR spectra of PP showed the development of absorption bands within the 1670–1800 cm^−1^ region, characteristic of carbonyl groups. This behavior is indicative of oxidation processes leading to polymer degradation (Xiong et al. 2017). Therefore, quantitative indicators such as the Carbonyl Index were used to systematically evaluate the changes in MPs after pretreatments in this study.

**Fig. 6:**
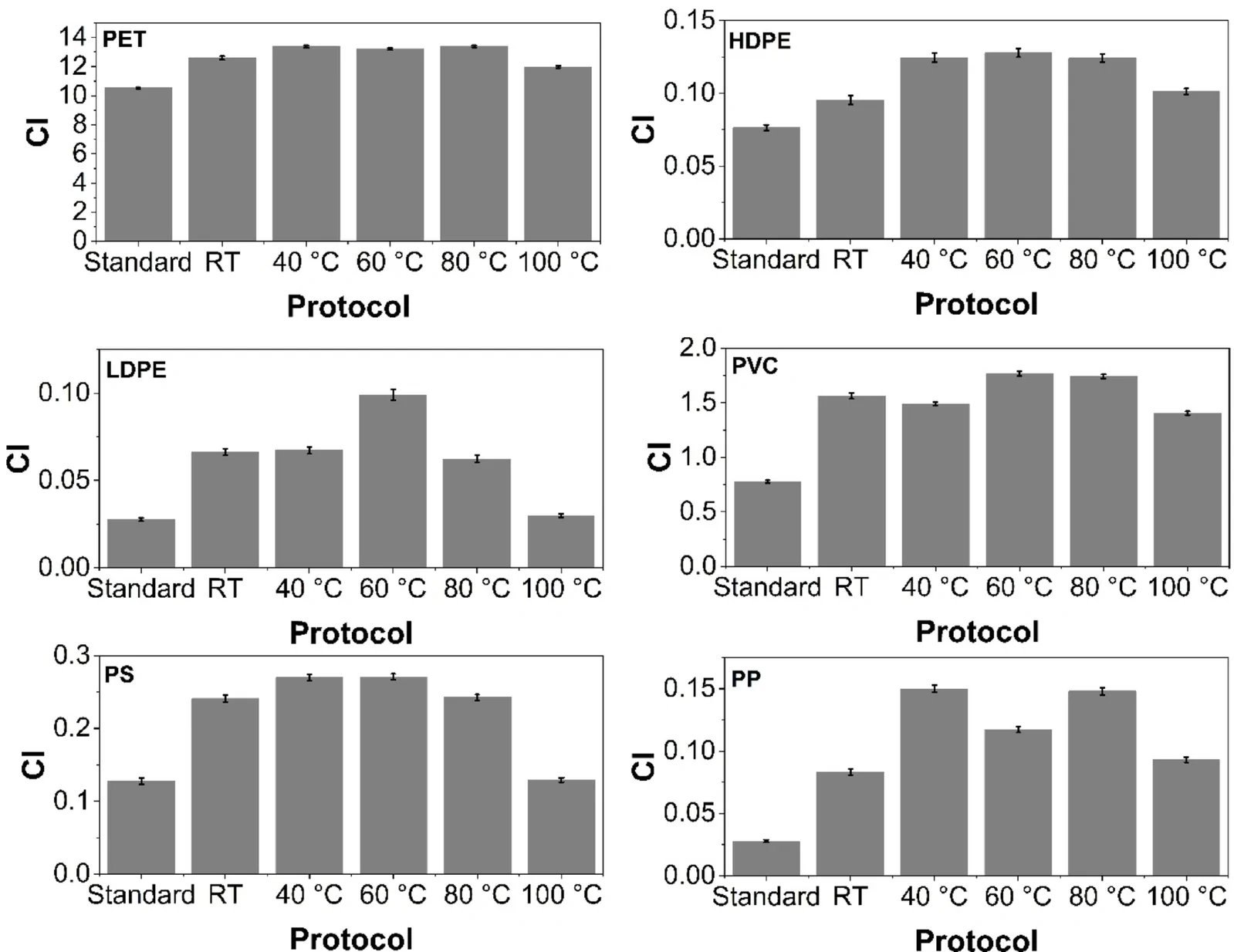
Changes in the CI of pure and oxidized polymers exposed to different oxidation protocols. Data are presented as mean ± standard deviation (SD) of three independent FTIR measurements (n = 3)

Figure 6 presents the results of calculating the CI for pure and oxidized polymers exposed to various oxidation protocols. Significant changes were observed in the carbonyl index of MPs after applying the Fenton digestion protocol compared with untreated MPs; however, these changes did not affect the structure or identification of the MPs studied. The CI data indicate an increase in carbonyl group formation with increasing temperature. The highest CI values were observed within the temperature range of 40–60 °C, suggesting moderate oxidative modification of the polymer surface. These results indicate that higher temperatures promote oxidation, which is reflected in increased carbonyl index values. In the PET, HDPE, LDPE and PS samples, the CI values obtained for protocol 5 were comparable to those of the control samples (pure polymer), indicating that, under extreme temperature conditions for 2 h, there was no oxidation of the polymers. These results corroborate with the argument that H_2_O_2_ decomposes rapidly at high temperatures (> 80 °C), thereby rendering oxidation ineffective (Bautista et al. 2007). Therefore, based on the results found, the optimal conditions for applying the Fenton reagent are a temperature between 40 and 60 °C and a reaction time of 2 h. These conditions demonstrated promising efficiency in digesting the organic matter present in MPs, without compromising their physicochemical integrity. These parameters provide an effective approach to degrading organic material associated with MPs, maintaining the viability of subsequent analyses and enabling reliable results in future studies.

### MPs Recovery

**Fig. 7:**
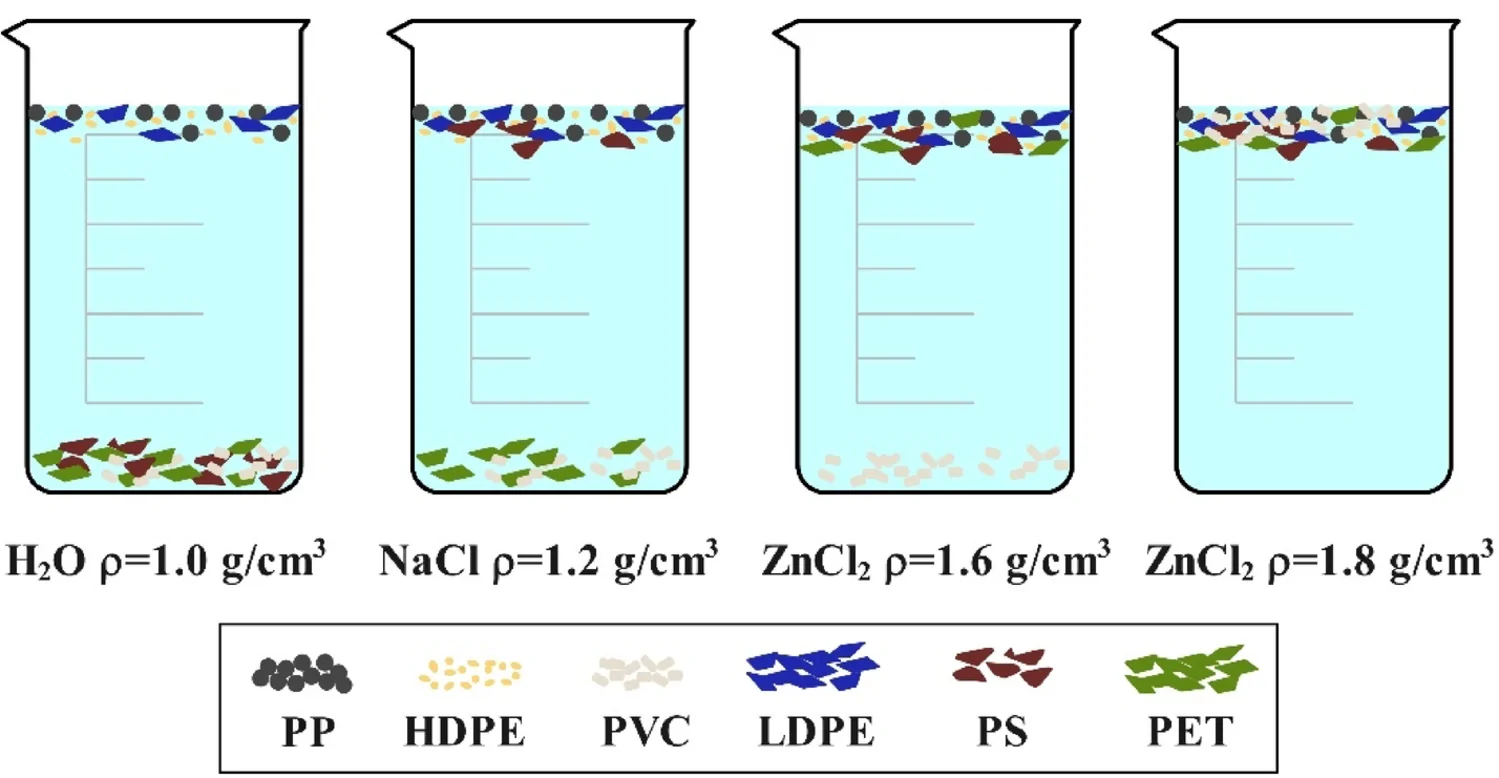
Density separation behavior of MPs in water (1.0 g cm⁻^3^), NaCl solution (1.2 g cm⁻^3^) and ZnCl₂ solutions (1.6 and 1.8 g cm⁻^3^). Data are presented as mean ± standard deviation (n = 3)

The results obtained using the different density media tested to determine the optimal conditions for the flotation of MPs particles from WWTP samples are presented in Table 4 and illustrated in Fig. 7. As shown in Table 4, water did not achieve good polymer separation efficiency, since it has a density (1.0 g.cm^−3^) lower than 50% of the polymers evaluated. Therefore, its use is not suitable for the extraction of MPs. Although saturated NaCl is suitable for the extraction of low-density MPs, it excludes denser polymers, such as PVC (1.10–1.45 g.cm^−3^) and PET (1.37–1.45 g.cm^−3^), being effective in the extraction of 67% of the plastics studied. Therefore, despite being inexpensive and chemically inert, the use of NaCl may lead to an underestimation of microplastic abundance, particularly for high-density polymers. For this reason, ZnCl_2_ has been used to quantify the abundance of MPs in several studies (Hidalgo-Ruz et al. 2012; Mattsson et al. 2022). In this study, which has higher densities (1.6–1.8 g cm^−3^), the polymers used to validate the separation method remained in suspension, enabling their effective extraction. Therefore, considering the achievable relative density of NaCl and from the results described in Table 4, ZnCl_2_ is considered the most appropriate saline solution for MP extraction.

**Table 4:** Behavior of polymers in density-based separation media

Separation medium (density)	Floating polymers	Sinking polymers
Water (1.0 g.cm^−3^)	PP, HDPE, LDPE	PET, PVC, PS
NaCl (1.2 g.cm^−3^)	PP, HDPE, LDPE, PS	PET, PVC
ZnCl₂ (1.6 g.cm^−3^)	PP, HDPE, LDPE, PS, PET	PVC
ZnCl₂ (1.8 g.cm^−3^)	PP, HDPE, LDPE, PS, PET, PVC	None

### Validation of the Proposed Methodology Using Real Wastewater Matrices

Following the optimization of the digestion and density separation procedures using reference MPs, the proposed methodology was validated using raw wastewater and dewatered sewage sludge collected from the Brasília North WWTP. These matrices were selected because they represent distinct levels of complexity commonly encountered in MPs monitoring studies. The two matrices presented markedly different organic matter contents. Raw wastewater presented an average COD of 882.7 ± 124.0 mg O_2_ L^−1^ (n = 3), whereas dewatered sewage sludge showed a substantially higher average COD of 30,600 ± 4,109 mg O_2_ L^−1^ (n = 3), confirming the greater organic load associated with sludge samples. The application of the optimized Fenton digestion protocol resulted in significant reductions in COD both matrices (Table 5). COD decreased to 165.4 ± 21.7 mg O_2_ L^−1^ in raw wastewater and to 5,420 ± 780 mg O_2_ L^−1^ in dewatered sludge, corresponding to removal efficiencies of 81.3% and 82.3%, respectively. These results demonstrate the effectiveness of the Fenton reagent in degrading organic matter and reducing matrix interference during subsequent analytical steps (Hurley et al. 2018; Motiejauskaitė and Barčauskaitė 2025).

**Table 5:** COD values before and after Fenton digestion

Matrix	Initial COD (mg O_2_ L^−1^)	Final COD (mg O_2_ L^−1^)	Removal (%)
Raw wastewater	882.7 ± 124.0	165.4 ± 21.7	81.3
Sewage sludge	30,600 ± 4,109	5,420 ± 780	82.3

**Fig. 8:**
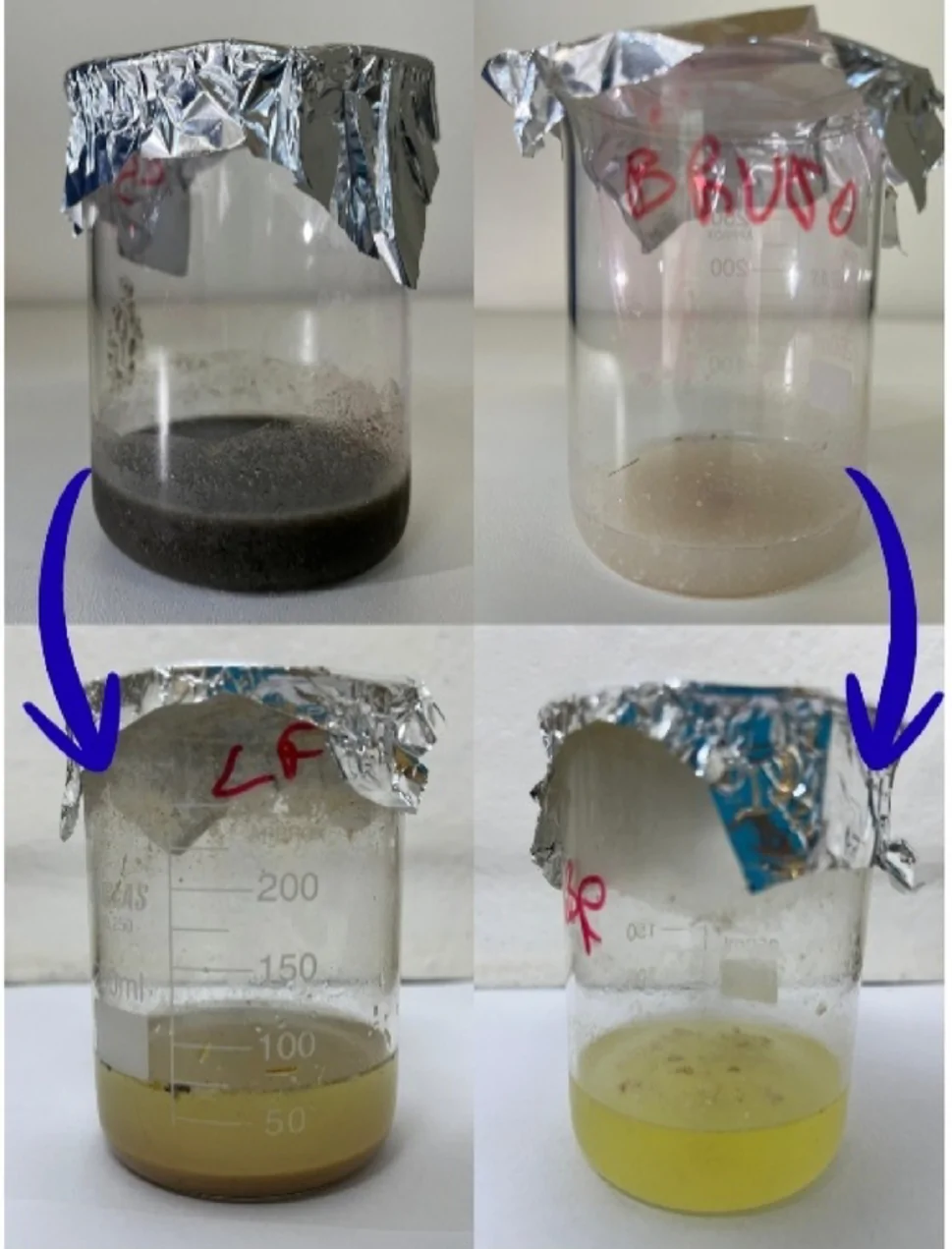
Visual appearance of raw wastewater and dewatered sewage sludge before and after Fenton digestion

In addition to the quantitative reduction in COD, significant visual changes were observed after digestion. Prior to treatment, the raw wastewater samples presented high turbidity and dark coloration due to the presence of suspended and dissolved organic matter. After the application of Fenton’s reagent, the samples became noticeably clearer and more translucent. Similar behavior was observed in the sewage sludge samples, although the degree of clarification was less pronounced due to their substantially higher organic content. The visual appearance of the samples before and after digestion is presented in Fig. 8. These observations qualitatively corroborate the COD results and demonstrate the strong oxidative capacity of the Fenton process.

For some sewage sludge samples, a single digestion cycle was insufficient to completely remove the visible organic matter. Residual suspended material remained after the first treatment, indicating that part of the organic matrix was resistant to oxidation under the applied conditions. In these cases, an additional digestion cycle was performed. After the second digestion, a marked reduction in suspended organic matter was observed, resulting in a more homogeneous suspension and significantly improving the conditions for density separation. These findings suggest that highly concentrated sludge samples may require successive digestion steps depending on their organic load and solids content. Following digestion, density separation using a ZnCl_2_ solution (1.8 g cm⁻^3^) enabled the successful flotation and recovery of all polymer types evaluated in this study, including PET and PVC, which are generally more difficult to recover due to their relatively high densities. The flotation of all spiked particles demonstrates the suitability of the selected density medium for the extraction of both low-density and high-density polymers from complex matrices (Han et al. 2019; Rodrigues et al. 2020).

**Fig. 9:**
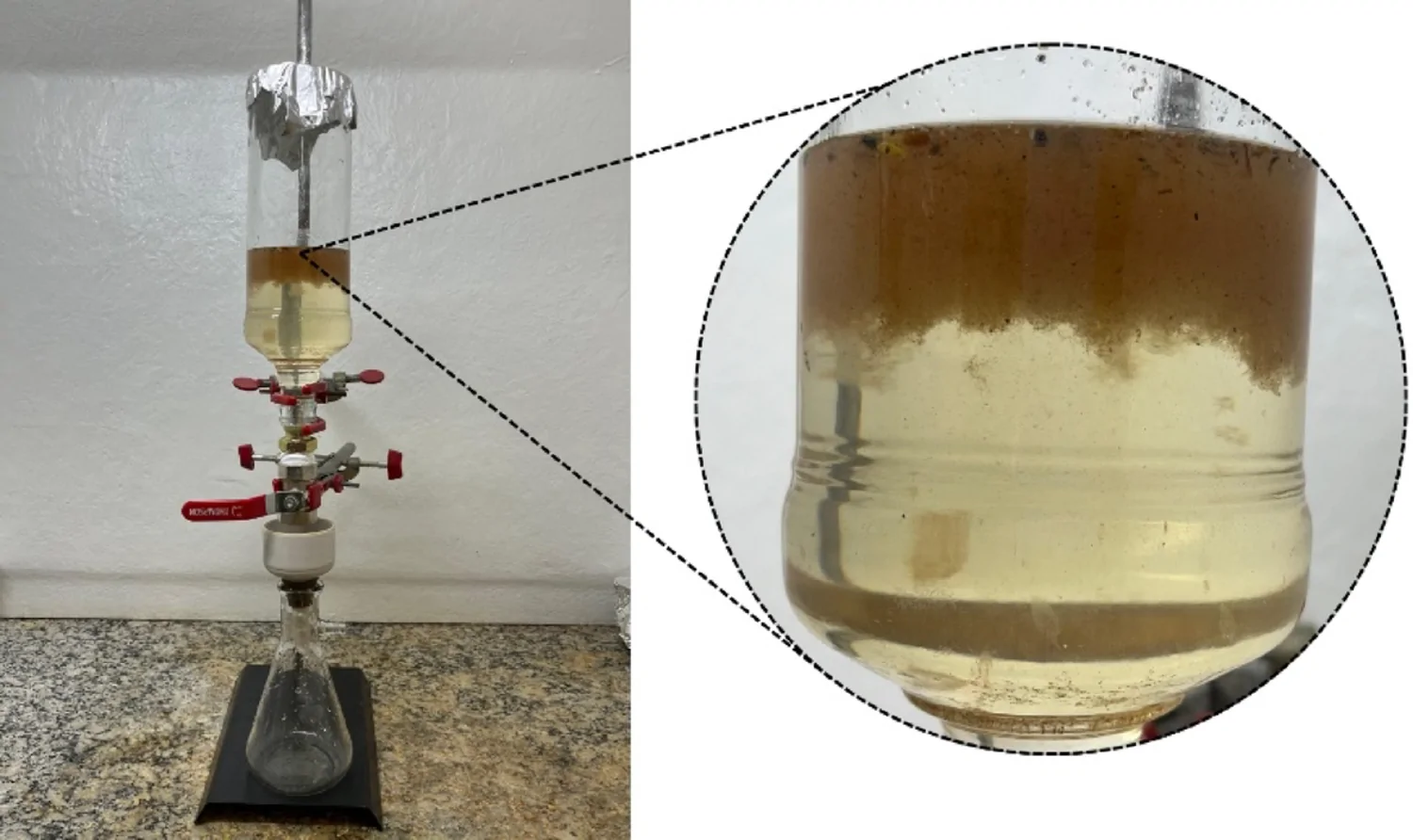
Density separation of MPs from wastewater-derived matrices using ZnCl_2_ solution (*ρ* = 1.8 g cm^−3^) in the custom separation unit. The image shows the formation of distinct phases after settling: the floating fraction, enriched in MPs, accumulates in the upper layer, while the sediment fraction, composed predominantly of residual organic matter and inorganic particles, accumulates at the bottom of the device

The efficiency of the density separation process is evident in Fig. 9, which shows the behavior of the digested wastewater-derived matrix after settling in the custom separation unit. A clear stratification of the sample was observed, with the floating fraction concentrated in the upper region of the device and the denser material accumulating at the bottom. The upper layer contained the MPs and lighter suspended material, whereas the lower fraction consisted predominantly of residual organic matter and denser inorganic particles. The formation of these distinct phases demonstrates the effectiveness of ZnCl₂ as a density medium and confirms its ability to promote efficient separation between MPs and the remaining matrix components.

The visual separation observed in Fig. 9 also corroborates the results obtained during the optimization experiments, in which ZnCl_2_ solutions presented superior performance compared with water and NaCl solutions, particularly for the recovery of high-density polymers such as PET and PVC. The use of a ZnCl_2_ solution with a density of 1.8 g cm^−3^ ensured that all target polymers remained in the floating fraction, thereby minimizing losses during sample processing and improving the reliability of subsequent identification procedures. The recovery of MPs from raw wastewater is particularly relevant because it demonstrates the applicability of the methodology under conditions representative of the influent of WWTPs, where MPs are directly exposed to suspended solids, organic matter, and microbial communities (Motiejauskaitė and Barčauskaitė 2025). Likewise, the successful extraction of MPs from dewatered sewage sludge confirms the robustness of the proposed methodology when applied to one of the most complex matrices generated during wastewater treatment (Thornton Hampton et al. 2023). Since dewatered sludge contains substantially higher concentrations of solids and organic matter than most liquid treatment streams, these results provide strong evidence that the methodology can be effectively applied throughout different stages of WWTPs.

Overall, the validation experiments demonstrated that the combination of Fenton digestion and ZnCl_2_ density separation provides an effective and reliable approach for MP extraction from wastewater-derived matrices. The high organic matter removal efficiencies achieved, the substantial visual clarification of the samples, the formation of distinct phases during density separation, and the successful recovery of all target polymers demonstrate the robustness of the proposed methodology. Furthermore, the successful application of the method to both raw wastewater and dewatered sewage sludge highlights its potential for routine monitoring of MPs throughout different stages of wastewater treatment systems and supports its use in future environmental assessments involving complex organic matrices.

### Application of the Validated Methodology to Native MPs

**Fig. 10:**
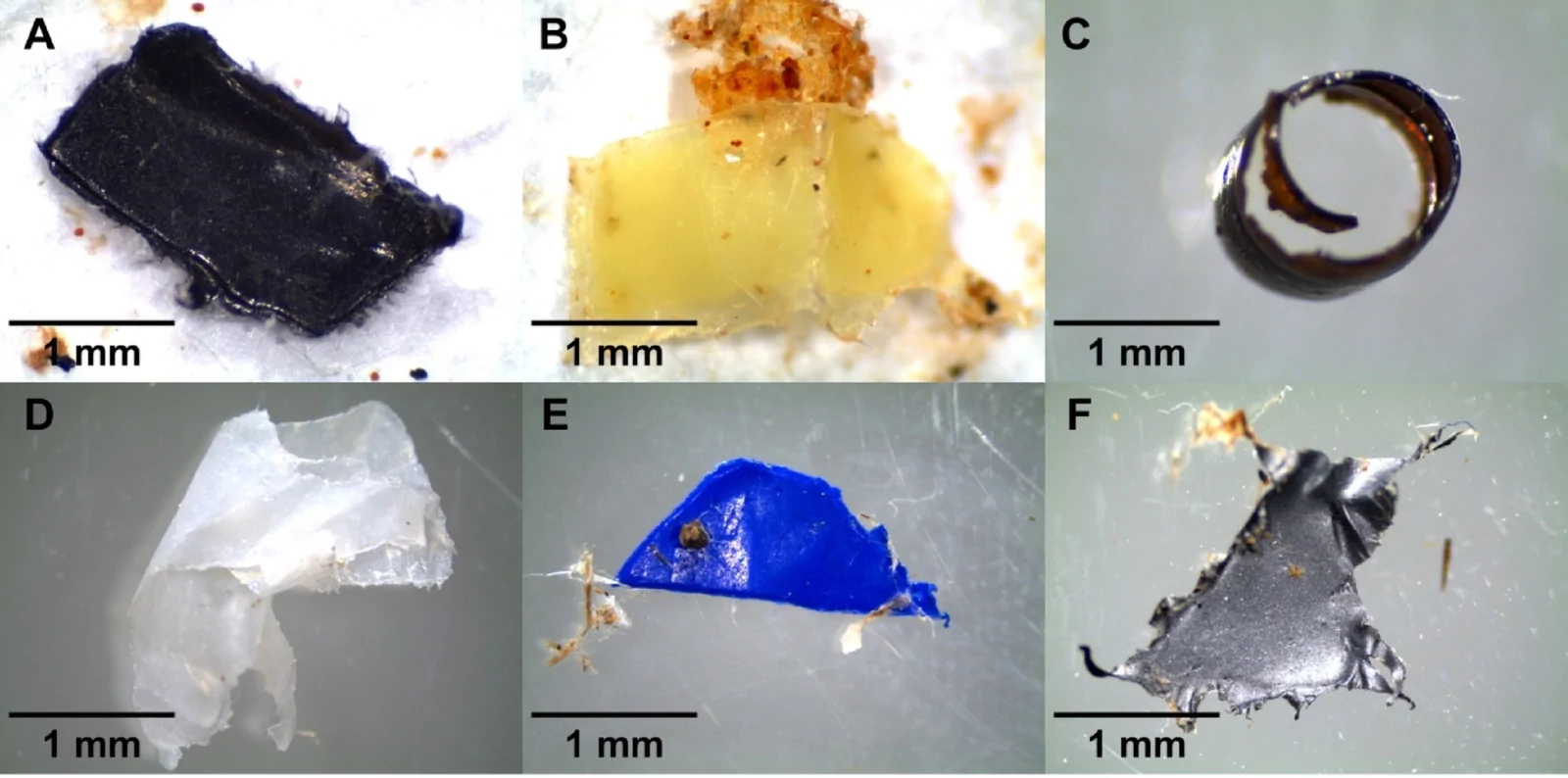
Representative stereomicroscope micrographs of native MPs recovered from wastewater treatment matrices at the Brasília North WWTP. Images A–C correspond to particles recovered from raw wastewater, whereas images D–F correspond to particles recovered from dewatered sewage sludge

Following validation experiments with reference microplastics, the optimized protocol was applied to raw wastewater and dewatered sewage sludge samples collected from the Brasília North WWTP to investigate the occurrence of native MPs under real-world operating conditions. MPs particles were recovered from both matrices after the combined digestion and density separation procedures. Representative stereomicroscope images are presented in Fig. 10. The recovered particles presented a wide range of morphologies, including fragments, films, and fibers, as well as variations in color, size, and surface texture. Such heterogeneity is characteristic of MPs found in wastewater treatment systems and reflects the diversity of plastic sources entering urban sewer networks.

Visual inspection revealed low levels of residual organic matter after sample processing, indicating that the optimized Fenton digestion protocol effectively reduced matrix interference. This aspect is particularly relevant for sewage sludge samples, which are typically characterized by high concentrations of organic matter, microbial biomass, and inorganic particles. The efficient removal of these constituents facilitated both the visual selection of particles and their subsequent spectroscopic characterization. The occurrence of MPs in both raw wastewater and dewatered sludge confirms that wastewater treatment plants act simultaneously as transport pathways and accumulation compartments for plastic particles. While part of the incoming MP load remains associated with the liquid phase, a significant fraction is retained in the sludge line via sedimentation and biological treatment. Consequently, sewage sludge is an important sink for MPs and may serve as a secondary source of environmental contamination when reused or improperly disposed of.

**Fig. 11:**
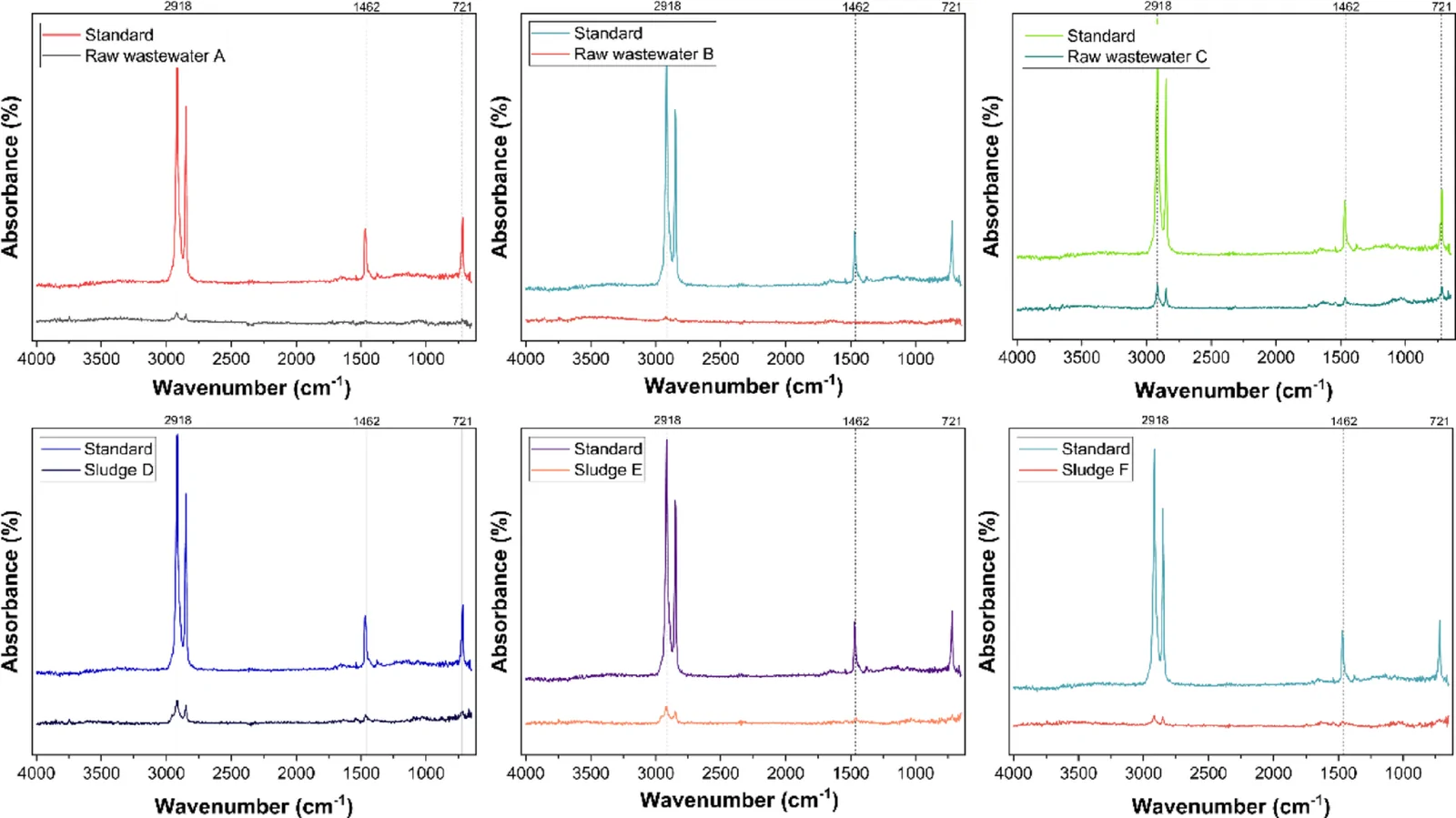
FTIR spectra of selected MPs particles. Images A–C correspond to particles recovered from raw wastewater, whereas images D–F correspond to particles recovered from dewatered sewage sludge

Representative particles recovered from raw wastewater and dewatered sewage sludge were analyzed by ATR-FTIR to confirm their polymeric composition and evaluate the applicability of the proposed methodology to environmental samples. The spectra obtained for the recovered particles are presented in Fig. 11. Among the identified polymers, polyethylene was the dominant material detected in both wastewater and sludge samples. Characteristic absorption bands were observed at approximately 2915, 2845, 1462, and 717 cm^−1^, corresponding to C–H stretching vibrations, CH₂ bending, and CH₂ rocking modes typically associated with polyethylene structures. The presence of an additional absorption band near 1377 cm^−1^ allowed the differentiation between LDPE and HDPE, as previously reported in the literature (Chércoles Asensio et al. 2009; Jung et al. 2018b). The spectra obtained from the recovered particles preserved the characteristic absorption bands required for polymer identification, indicating that the pretreatment procedures did not compromise spectroscopic analysis. This observation is consistent with the results obtained during the optimization stage, where FTIR and CI analyses demonstrated that the Fenton digestion protocol promoted only limited surface oxidation without altering the polymeric fingerprints necessary for reliable identification.

## Conclusion

This study demonstrated that reference microplastics can be reliably produced from commercially available plastic materials, such as HDPE, LDPE, PVC, PP, PS and PET. Characterization by ATR-FTIR revealed that these reference MPs maintained absorption patterns characteristic of each polymer, facilitating their identification. Furthermore, chemical digestion protocols, characterized by the Fenton reaction, did not compromise the identification of the polymers by ATR-FTIR. However, the resistance of the polymers to the oxidation protocols varied with temperature, with an increase in carbonyl adsorption observed at higher temperatures. However, the tested polymers did not show significant changes in their carbonyl index after the application of the protocols, suggesting a general resistance to oxidation under these conditions. In the density separation step, the ZnCl_2_ saline solution demonstrated high efficiency for the extraction of MPs, especially for denser polymers such as PVC and PET. However, despite its analytical effectiveness, factors such as toxicity, higher operational cost, and disposal requirements should also be considered when selecting this saline solution for routine applications. Validation experiments performed using raw wastewater and dewatered sewage sludge confirmed the applicability of the proposed methodology under realistic environmental conditions. The optimized protocol promoted substantial reductions in organic matter content, facilitated the separation of MPs from complex matrices, and enabled the successful recovery and identification of both reference and native microplastics. The predominance of polyethylene-based polymers in the analyzed samples is consistent with the widespread use of these materials in urban environments and with findings reported in previous studies. Overall, the combined use of Fenton digestion, ZnCl_2_ density separation, stereomicroscopy, and ATR-FTIR analysis proved to be a robust and reliable analytical approach for microplastic extraction and characterization in wastewater-derived matrices. The methodology demonstrated strong potential for application in monitoring programs aimed at evaluating the occurrence, transport, and fate of microplastics throughout wastewater treatment systems. Furthermore, the results contribute to the ongoing development of standardized protocols for microplastic analysis in complex environmental matrices.

## Data Availability

The datasets generated and/or analyzed during the current study are available from the corresponding author on reasonable request.
